# See What You Feel: A Crossmodal Tool for Measuring Haptic Size Illusions

**DOI:** 10.1177/2041669520944425

**Published:** 2020-08-10

**Authors:** Olga Daneyko, Angelo Maravita, Daniele Zavagno

**Affiliations:** Department of Psychology, Sociology and Politics, Sheffield Hallam University; Department of Psychology, University of Milano-Bicocca, Milan, Italy

**Keywords:** haptics/touch, visuo-haptic interactions, Uznadze haptic aftereffect, crossmodal matching

## Abstract

The purpose of this research is to present the employment of a simple-to-use crossmodal method for measuring haptic size illusions. The method, that we call *See what you feel*, was tested by employing Uznadze’s classic haptic aftereffect in which two spheres physically identical (test spheres) appear different in size after that the hands holding them underwent an adaptation session with other two spheres (adapting spheres), one bigger and the other smaller than the two test spheres. To measure the entity of the illusion, a three-dimensional visual scale was created and participants were asked to find on it the spheres that corresponded in size to the spheres they were holding in their hands out of sight. The method, tested on 160 right-handed participants, is robust and easily understood by participants.

## Introduction

The purpose of this research is to present the employment of a crossmodal matching method that we employed to quantify a rather strong haptic aftereffect.

An aftereffect is a phenomenon that can be described as changes in the perception of a present stimulus due to a prior event or activity, without conscious guidance or intentions. For instance, in the haptic aftereffect first described and studied by Uznadze (1930, 1966), two spheres physically identical (test spheres) appear different in size after that the hands holding them underwent an adaptation session with other two spheres (adapting spheres), one bigger and the other smaller than the two test spheres: the test sphere held in the hand adapted to the big adapting sphere appears smaller than the test spheres held in the hand adapted to the small adapting sphere. To explain the illusion, Uznadze conceptualized a *psychological set* that he described as a special psychological state of *readiness* for a given action and as a dynamic relationship between the individual and the environment. According to Uznadze, this phenomenon is rooted both in the participants’ previous experience and in their expectations. In Uznadze’s Theory of Set, human behavior is conceptualized as survival in an ever-changing environment. It describes complex interactions that can provide insight into how and the extent to which external influences affect human behavior, in particular physiological and mental states. According to Uznadze, in order to effectively navigate through the environment, the human brain creates sets, which function as a collection of subconsciously preplanned expectations and behaviors for a specific situation, driven by contingent experience.

The *Uznadze effect* (Piaget & Lambercier, 1944) appears in several sensory modalities and has been studied systematically by Uznadze himself and his students (Nadirashvili, 1978; Prangishvili, 1967) and also by a number of scientists who attempted to understand the nature of such phenomena and the mechanisms driving them (Takai, 1984; Walker & Shea, 1974; Zinchenko, 1978).

As with all illusions, a key to understanding the haptic Uznadze size-contrast illusion also requires the ability to measure its magnitude as relevant factors are manipulated. In general, studying perceptual illusions requires the ability to measure how the illusion is affected by the modulation of certain physical parameters within a controlled setup (e.g., Daneyko et al., 2014; Gallace et al., 2012; Zavagno et al., 2017). This is, however, a tricky business. First, no psychophysical measurement should be considered as an absolute value of an illusion’s size, as the measurement method employed can affect the magnitude outcome, its representation, and even its interpretation (Zavagno et al., 2010, 2018).

Measurements of perceptual phenomena have validity within an experimental design and across experiments, given that the parameters that govern the measuring instrument or method are kept constant across conditions and experiments (Daneyko et al., 2010; Zavagno et al., 2010, 2018). While the emerging results are important for determining factors relevant to the illusion, they are only gross indicators of the phenomenon’s magnitude modulations. This said, defining a proper method for measuring the Uznadze haptic aftereffect is rather difficult, given the sensory modality in which it appears. In fact, most of the studies pertaining to the haptic illusion (e.g., Cummins, 1976; Tchanturia et al., 2001) employed a qualitative measurement method. This consists of asking the participant which of the two test objects is bigger after haptic adaptation has been completed. This method, which was employed by Uznadze himself for all sensory modalities and perceptual continua he investigated, does not measure the size of the aftereffects; it only allows for stating the presence of the phenomenon and determining its direction. That is, whether the phenomenon is one of contrast or of assimilation. In other words, considering the haptic modality with spheres, whether the test sphere held in the hand adapted to the small adapting sphere appears bigger whilst the test sphere held in the hand adapted to the big adapting sphere appears smaller (*contrast* with the adapting spheres), or vice versa (*assimilation* with the adapting spheres).

We are aware of only a few studies that attempted quantitative measurements of Uznadze types of aftereffects. Piaget and Lambercier (1944) and Khachapuridze (1962) studied the Uznadze effect in the visual modality by employing a matching method in which, after visual adaptation to two adapting disks, an experimenter gradually modified the size ratio between two test spheres until participants perceived them as equal. More recently, Kappers and Bergmann Tiest (2013) conducted experiments on the haptic size aftereffect via a bisection task. They confirmed the direction of the phenomenon reported in the previous literature (Maravita, 1997; Uznadze, 1966); moreover, their method allowed for estimating the magnitude of the illusion at a 16% difference in the size of a test object after adaptation to bigger and smaller objects of the same shape. Kappers and Bergmann Tiest (2014) also showed that if the test objects do not share the same shape as the adapting objects, then the magnitude of the illusion decreases.

Before introducing the crossmodal matching method we applied, it is important to have a closer look to the only other psychophysical method employed to study the Uznadze haptic size-contrast aftereffect.

The bisection task devised by Kappers and Bergmann Tiest entails adapting both hands to two test spheres different is size. Placed on top of thin rods, the test spheres could be grasped by blindfolded participants, thus bypassing differences in weight due to differences in size. After adaptation, one hand was removed from an adapting sphere and guided by the experimenter to a test sphere. The participant then had to state whether the test sphere was bigger or smaller than a (hypothetical) sphere that would be of midway size between the two adapting spheres. If, for instance, the participant evaluated the test sphere to be smaller, the next test sphere would be 1 cm^3^ bigger than the previous test sphere. After an evaluation, the hand would be guided back to the adapting sphere, and a new test sphere would replace the previous one, and the same procedure would be repeated (for a detailed account of the method, see Kappers & Bergmann Tiest, 2013).

The method provides a way of measuring the Uznadze haptic aftereffect within a same sensory domain, as in the studies by Piaget and Lambercier (1944) and by Khachapuridze (1962), who however studied the visual size aftereffect. Moreover, Kappers and Bergmann Tiest’s method, which derives from a previous work on haptic volume and area perception (Kahrimanovic et al., 2010), has the advantage of nullifying the effect that weight might have on haptic size perception. Why then are we presenting another method? The bisection method has some critical aspects that we think need to be considered.

First, while both hands are adapted to an adapting sphere, only one hand is engaged with a test sphere during the size evaluation process, while the other is still grasping an adapting sphere. It is unknown how this aspect of the procedure may impact on an aftereffect that relies on simultaneous presentations. Experiments are required to clear this issue.

The second aspect is critical: Participants are asked to determine whether a test sphere is bigger or smaller than the mental representation of a sphere, which size should be the midpoint of the size interval between the small and the big adapting sphere. This is problematic because the hypothetical midpoint sphere is not actually experienced by the participant, it is imagined. This constitutes an additional task, for which there is no control, that adds to the size evaluation task. Moreover, the midpoint sphere is imagined while one hand is grasping an adapting sphere and the other a test sphere. This also raises a few issues. First, the mental representation of the midpoint sphere is carried out at a high cost in terms of cognitive resources: The participant is grasping only one of the adapting spheres, while the other hand is grasping the test sphere; this means that the participant must work out the size of the midpoint sphere by combining an actual haptic experience with the memory of a haptic experience. Is the mental representation of the midpoint sphere affected by what the hands are grasping? For instance, does it stay constant or is it affected by the size interval that separates the test sphere from the adapting sphere that is still being grasped by the participant? In other words, how good are participants in imagining the midpoint sphere, and how stable is this mental representation across evaluations? The sum of the aforementioned issues might constitute a threat to the validity of the measurement method.

As we were interested in investigating the Uznadze haptic aftereffect, we wanted to devise a measurement method that would be understood by participants and replicable across experiments. The method in question is crossmodal, as it requires matching the size of a haptic object to the size of a visual object embedded within a visual three-dimensional scale. We therefore called the method *See what you feel*. The rich literature on vision and haptic interactions includes studies in which the perception of a same object in the two sensory modalities is compared (e.g., Gepshtein et al., 2005; Klatzky et al., 1987; Norman et al., 2012; Rock & Victor, 1964). In our study, however, we are not interested in such comparisons or in the multisensory interactions between vision and haptics per se; instead, we are testing the feasibility of employing one sensory modality (vision) to measure the strength of an illusion that occurs in another sensory modality (haptics).

Experiments were conducted in the traditional Uznadze fashion, but instead of asking which test sphere appeared bigger (or smaller) after adaptation, we asked participants to find the spheres that visually matched their haptic sensations of size on a set of spheres placed in front of them, organized left to right from the smallest to the biggest (the visual scale). The procedure, while simple for participants to carry out, introduces some questions that we addressed experimentally. These are as follows:

- How good are participants in evaluating the size of the test sphere before adaptation? To study the issue each participant evaluated, one hand at a time, the size of the test sphere prior to adaptation, that is, during a pretest phase. We also asked them to evaluate the sizes of the two adapting spheres.
- Given the orientation of the visual scale from small-to-big (left to right), would it make a difference whether the small adapting sphere (which normally determines a test sphere to appear bigger) was placed in the left hand versus the right hand? To study this question, half of the participants (80) were adapted to the small adapting spheres with their right hand, the other half were adapted to the small adopting sphere with their left hand.
- Size evaluations are necessarily sequential, meaning that a few seconds pass between the evaluation of the size of the test sphere held in one hand and the size of the test sphere held in the other hand. Will the sequence affect judgments because of the time elapsed between the two matchings? To study this, half of the participants (80) started evaluating the sizes of the test spheres from their right hand to their left hand; vice versa, the other half started evaluations from left to right.
- Finally, how does the distance of the visual scale impact on size evaluations? To test this, for half of the participants (80), the matching scale was placed at a distance of 30 cm (near), for the other half, it was placed at 160 cm (far).

## The Experiment

### Participants

Participants were 160 people from the University of Milano-Bicocca (76 males, mean age = 35) randomly divided into two groups of 80 distinguished by the distance of the matching scale (near; far). The participants from each group were then randomly assigned to one of the four experimental conditions. Each participant was asked whether they were right-handed, a condition that was requested to participate in the experiment, and that was then tested with the Edinburg Inventory (Oldfield, 1971). All participants were naive to the purpose of the experiment and not familiar with the Uznadze haptic size aftereffect. Prior to taking part in the experiment, all participants completed an informed consent with an overview of the experimental procedure.

### Materials

**Figure 1.**
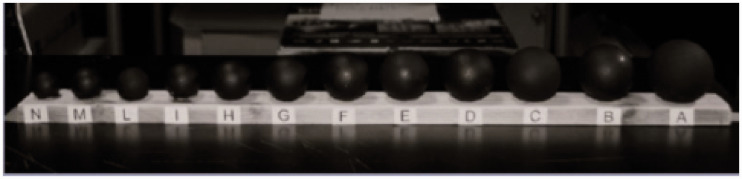
The Visual Scale.

The measuring instrument was a physically three-dimensional scale made of 12 spheres organized in growing order from left to right, mounted on a wood base that measured 60 cm in length. Each sphere was denoted by a capital letter printed on the base directly under the sphere. The sizes of the spheres are indicated in terms of their diameter and were the following: 2.2 cm (N), 2.5 cm (M), 2.7 cm (L), 2.9 cm (I), 3.1 cm³ (H), 3.5 cm (G), 3.8 cm (F), 3.9 cm (E), 4.1 cm (D), 4.3 cm (C), 4.5 cm (B), and 4.7 cm (A). The spheres were painted matt black and fixed at a distance of 2 cm from each other’s horizontal diameter (Figure 1).

Adapting stimuli (AS) and test stimuli (TS) were spheres made of celluloid (the same material with which table tennis balls are made); average weight gr 2.7 ± 0.5. The small AS measured 2.9 cm in diameter (letter I on the scale); the big AS measured 4.3 cm in diameter (letter C on the scale); and TS measured 3.9 cm in diameter (letter E on the scale).

A narrow worktable was used during the experiment that allowed participants to put their arms in an empty compartment under the tabletop, open on both sides, which blocked their hands from sight during the different phases of the experiment.

### Experimental Design

Like other studies on aftereffects (e.g., Bruno et al., 2018; van Dam et al., 2016; van der Horst et al., 2008; Vogels et al., 1996, 1997, 2001), we employed a series of standardized phases, which in our case were as follows: (a) a pretest phase, (b) an adaptation phase, and (c) a test phase. Sphere matchings between the haptic sensation and the spheres on the visual scale were required for Phases 1 and 3.

Purpose of the pretest phase was to determine for each participant the visual scale value of each sphere experienced haptically by both hands prior to adaptation.

Purpose of the adaptation phase was to create the conditions for the illusion to occur by having participants clench the spheres. For this phase, adapting spheres were placed in the hands of a participant, who was instructed to clench them, then the participant was asked to open the hands and the spheres were removed to be placed right after again in the participant’s hands, who was asked to clench again. This sequence was repeated for exactly 15 times. On the 16th time, without any warning, the two adapting spheres were replaced by the two test spheres, and the participant was asked to clench them and to find a match for the two spheres on the visual scale (test phase). This type of procedure has been adopted in several studies who employed the Uznadze haptic illusion as it allows to avoid disruption between the adaptation and the test phases (e.g., Cummins, 1976; Tchanturia, Serpell, Troop, & Treasure, 2001).

For the pretest phase, the within-subject factor was the *Hand* (left, right) clenching the sphere that was being matched on the visual scale; the between-subjects factors were *Scale Distance* (30 cm; 160 cm), and the hand from which participants began the matching sequence (*First Hand*, left, right).

For the test phase, the within-subjects independent variables were the size of the two adapting spheres (factor *Adapting*: small, big) and *Hand*. The first between-subjects factor was *Scale Distance*, which determined two groups of participants, each made of 80 participants. The other between-subjects factors were *First Hand* and the *Position* of the small adapting sphere (left hand; right hand); the combination of these two factors determined four subgroups for each of the two scale distances.

### Procedure

After reading and signing the informed consent form, participants self-compiled the simplified Edinburg inventory form. The experiment consisted of three phases, indicated as follows: pretest, adaptation, and test. During these phases, participants sat at the experimental table and placed their arms in the two-sided open compartment under the table top so that their hands rested out of sight with their palms facing upward.

During the pretest phase, participants evaluated the size of the test sphere (TS, 3.9 cm) and that of the two adapting spheres (AS, 2.9 and 4.3 cm). The three spheres were evaluated in sequence twice, one hand at a time, always starting with the test sphere (as this was the measure we were interested in); the adapting spheres were presented in random order. The sequences were repeated alternating hands. The first evaluation series always started from the hand that characterized the participant’s group (factor *First Hand*).

During the adaptation phase (see *Experimental Design* section), the small adapting sphere was always placed in the same hand, according to the participant’s group (factor *Position*). Immediately after the last adaptation sequence, the adapting spheres were replaced by the two tests spheres. The participant was asked to clench hands and to find a match for the two test spheres on the visual matching scale placed in front of them at one of the two distances. The matching task was conducted starting from the hand that characterized the participant’s group (factor *First Hand*).

The whole experimental session, including handedness determination and participant debriefing, lasted approximately 30 minutes.

### Results

The experiment produced two sets of data: evaluations of the test sphere’s size for both hands prior to adaptation and after adaptation. The first set of data, that we identified as pre*T*, quantifies the difference between the visually evaluated size of the haptically perceived test sphere and its actual size for each of a participant’s hand.

The second set of data concerns similar visual size matchings but after adaptation (see Supplemental Figure 1). To measure the effect of adaptation on the test spheres (i.e., the size of the aftereffects for the two hands), we need to consider the difference between the visual size evaluations of a test sphere for a certain hand prior to adaptation and after adaptation. We therefore subtracted pre*T* from *T*, deriving the value Δ*T* (Δ*T* = *T* − pre*T*), which is a measure of the visually perceived distance between *T* (perceptual evaluation of a test sphere TS size after adaptation) and pre*T* (perceptual evaluation of a test sphere TS prior to adaptation). For each participant, there were therefore two Δ*T* values, one corresponding to the size impression after adaptation to the small sphere (Δ*T_s_*), the other corresponding to the size impression after adaptation to the big sphere (Δ*T_b_*).

#### Pre T data

**Figure 2.**
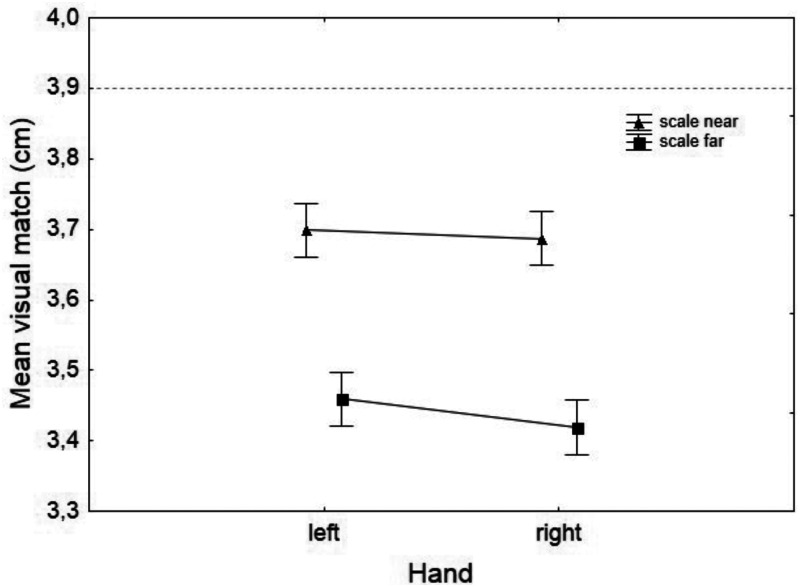
Mean Visual Matches for TS Before Adaptation. Numeric values correspond to the diameters of spheres. The dashed line corresponds to the actual diameter of a test sphere. Error bars are standard errors. One sample *t* tests show that mean visual matches differed statistically from the test sphere’s actual size (see Table 1).

Figure 2 shows mean results for the pretest on the test sphere distinguished for *Hand* and *Scale Distance.* An analysis of variance (ANOVA) for repeated measures was carried out on the data with *Hand* as within-subject factor and *Scale Distance* and *First Hand* as between-subjects factors. *Hand* and *First Hand* did not determine significant main effects (*p* = .256 and *p* = .459, respectively); *Scale Distance* determined a significant main effect: *F*(1, 156) = 26.734, *p* < .001, η^2^_p_ = .146. None of the interactions were statistically significant. One sample *t* tests were carried out on the pretest data for each hand and each scale distance; a significant difference between the actual diameter of the test sphere (3.9 cm) and its perceived size was found (see Table 1).

**Table 1.** One Sample *t* Tests Results for the Visual Match of the Test Sphere Compared With Its Actual Size.

Hand and scale	Mean	Standard deviation	*t*_79_	*p*
Left near	3.70	0.34	−6.6	.001
Right near	3.69	0.35	−6.6	.001
Left far	3.46	0.34	−12.7	.001
Right far	3.42	0.33	−14.2	.001

To understand whether the underestimation found with TS is something that characterizes this type of crossmodal size estimation method, we also conducted two ANOVAs for repeated measures on the size matchings for the two ASs, with *Hand* as within-subject factor and *Scale Distance* and *First Hand* as between-subject factors. Again, *Hand* and *First Hand* did not determine significant main effects on visual size evaluations (*p* > .1), while *Scale Distance* did: small sphere, *F*(1, 156) = 5.95, *p* < .05, η^2^_p_ = .037; big sphere, F(1, 156) = 5.31, *p* < .05, η^2^_p_ = .033. As with the test sphere, both adapting spheres were underestimated with respect to their actual sizes (respectively, 2.9 and 4.3 cm), as confirmed by one sample *t* tests (see Table 2).

**Table 2.** One Sample *t* Test Results for the Visual Match of the Adapting Spheres Compared With Their Actual Sizes.

Adapting sphere, hand and scale	Mean	Standard deviation	*t*_79_	*p*
Small, left, near	2.39	0.22	−20.5	.001
Small, right, near	2.40	0.21	−20.7	.001
Small, left, far	2.46	0.26	−15.0	.001
Small, right, far	2.50	0.25	−14.1	.001
Big, left, near	3.91	0.29	−11.5	.001
Big, right, near	3.90	0.36	−10.0	.001
Big, left, far	4.03	0.34	−7.09	.001
Big, right, far	4.01	0.36	−7.11	.001

#### Test Data

An ANOVA for repeated measures was carried out on the Δ*T* data to measure the effect of adaptation on the TS with *Adaptation* (Δ*T_s_*, Δ*T_b_*) as within-subject factor, and *Scale Distance* × *First Hand* × *Position* as between-subject factors (2 × 2 × 2). Of all factors considered, only *Position* did not determine a significant main effect (*p* = .186) or significant two-way interactions with the other variables.

**Figure 3.**
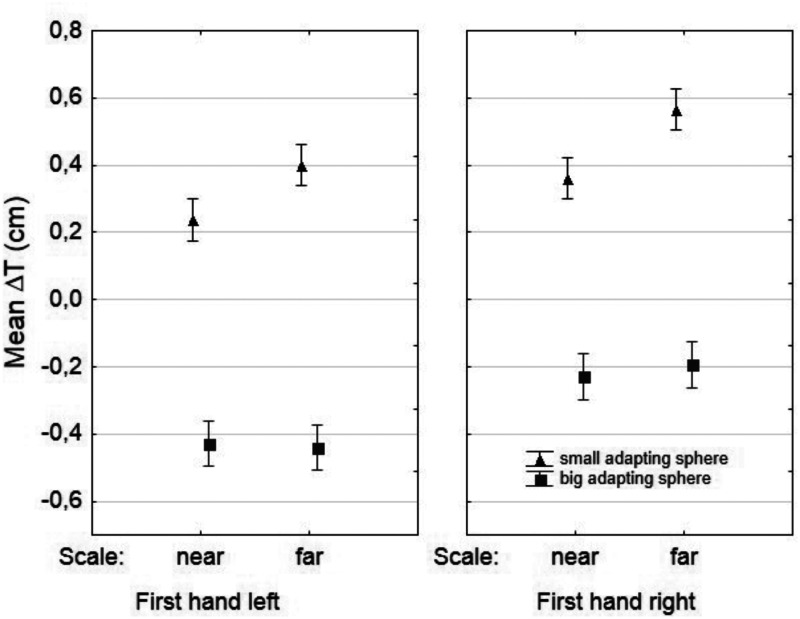
Results for TS After Adaptation Expressed as a Difference Between the Size Evaluations of TS After and Prior to Adaptation (ΔT, See Main Text). Error bars are standard errors.

Figure 3 reports mean Δ*T* values in relation to factors *Adaptation* × *First Hand* × *Scale*. As expected, *Adaptation* induced a significant main effect, *F*(1, 152) = 238.073, *p* < .001, η^2^_p_ = 0.61, along with *Scale* and *First Hand* (respectively), *F*(1, 152) = 9.463, *p* < .005, η^2^_p_ = 0.0586 and *F*(1, 152) = 23.272, *p* < .001, η^2^_p_ = 0.132. The interaction *Adaptation* × *Scale* was also significant: *F*(1, 152) = 10.312, *p* < .005, η^2^_p_ = 0.063, while the other two-way interactions were not significant (*p* > .5).

Unpaired *t* tests with *Scale* as grouping factor allowed us to determine that scale distance affected significantly only the evaluation of the test sphere in the hand adapted to the small adapting sphere, $\bar{\Delta T}$*_s_*_-near_ = 0.195, $\bar{\Delta T}$*_s_*_-far_ = 0.482, *t*(158) = 4.290, *p* < .001, which was matched with a bigger sphere on the scale placed at a greater distance. Scale distance had no effect on the match for the test sphere held in the hand adapted to the large adapting sphere (*p* = .858).

As seen in Figure 3, first hand determined a slight shift up the matching scale when it was the right hand, hence for the two scale distances, we calculated the size of the illusion as the difference between $\bar{\Delta T}$*_s_* and $\bar{\Delta T}$*_b_*. The overall magnitude of the illusion is given by the ratio $\bar{\Delta T}$*_s_*$\;-\;\bar{\Delta T}b/\bar{\mathrm{Pre}T}$, which results in a 17% departure from the mean visual match of the test sphere prior to adaptation for the condition near (scale at 30 cm) and a 23% departure for the condition far (160 cm). A two-way ANOVA was conducted on the Δ*T_s_* − Δ*T_b_* data with *First Hand* and *Scale Distance* as independent variables. Only *Scale Distance* determined a significant effect: *F*(1, 156) = 4.016, *p* < .05, η^2^_p_ = 0.025. *First Hand* and the interaction *First Hand* × *Scale Distance* were not significant (*p* > .3 and *p* > .9, respectively).

### Discussion

We posed four questions regarding the *See what you feel* method that we can now answer.

First: How good are participants in evaluating visually the tactual perception of the size of the test spheres before adaptation? The pretest data collected for all three spheres employed in our experiment—the test sphere and the two adapting spheres—show an underestimation of the visual size of the spheres clenched out of sight. This appears to be a feature of the present crossmodal matching method, and something that should be further addressed empirically.

Second: Does the position of the adapting spheres (which hand holds which) affect the illusions, given that we kept the orientation of the visual scale constant? To test this, we had 80 participants adapted to the small sphere in their left hand, and the other 80 adapted to the small sphere in the right hand (factor *Position*). The adaptation to the small sphere commonly produces an overestimation of the test sphere, while adaptation to the big sphere produces an underestimation of the test sphere. Therefore, 80 participants were evaluating the test spheres after adaptation feeling a smaller test sphere in their left hand and a bigger one in their right hand, in accordance with the direction of the scale. Of these, 40 participants actually started test evaluations with their left hand (factor *First Hand*). *Position*, however, did not determine main effects on visual size evaluations, nor were its two-way interactions with the other variables significant. This result is important because it answers another question: Are right handers better in evaluating objects held in their right hand? Our results do not suggest such a positive bias.

Third: After adaptation, participants were asked to judge first the size of one test sphere and then of another. Did the sequence of test sphere evaluations affect judgments because of the time elapsed? We found a significant main effect for the factor *First Hand*, but no significant two-way interactions with the other factors. The graph in Figure 3 does not show an *advantage* in size evaluations in reference to *First Hand*; it rather suggests a scale shift: When starting with the right hand, right handers tend to slightly upscale their evaluations. There is not, however, an advantage in terms of illusion size. This finding is consistent with a lack of effect of the factor in the pretest data.

Fourth: Does the distance of the visual scale impact on visual size evaluations of haptic objects? This question is interesting per se, as it concerns the mapping of dimensions from one sensory modality to another. In relation to the new method we are testing, we found an effect of visual scale distance, which, however, seems to be confined to the evaluation of TS held in the hand adapted to the small sphere, as specified by the interaction *Adaptation* × *Scale Distance*. Furthermore, an effect of scale distance was found for all three spheres with the pretest data. As far as we know, this effect of scale distance on haptic size evaluation has never been reported and bears no resemblance to the size-weight illusion (Ellis & Lederman, 1993). To better understand the effect of scale distance on haptic size evaluations, more experiments need to be carried out.

Finally, despite noticeable methodological differences, the magnitudes of the haptic aftereffect illusion we report are remarkably similar to the strength reported by Kappers and Bergmann Tiest (2013; 16%), in particular for our condition near (17%). This strongly suggests that the two methods are measuring the same thing.

## Conclusions

Measuring the size of aftereffects is a tricky business, in particular, because the tendency is to measure aftereffects in the same sensory domain in which they occur, which often requires the setup of rather complex methods to capture the actual size of an illusion. However, the ability to measure the magnitudes of such illusions is necessary if we want to study their dynamics and the factors that modulate them. The crossmodal method *See what you feel* is rather simple to employ and altogether elegant, allowing to measure in a reliable way the size of a complex haptic aftereffect, such as the Uznadze illusion, without having to devise a way of bypassing the effect of the illusion itself on the measurement task. The simplicity of the method means that, as with all matching methods (Zavagno et al., 2010; Zwislocki, 1991), participants understand the task quite easily and just as easily they carry it out. The fact that the magnitudes we found are in line with those reported by Kappers and Bergmann Tiest (2013) is further support to the validity of the method. The simplicity of the method also means that it can be easily employed with children, and that it can probably become a useful tool to measure senior participants’ sensitivity to the illusion. This usability aspect with people of all ages is interesting as the Uznadze haptic illusion has been and can be employed to study different psychological aspects, for instance, in relation to eating disorders (e.g., Tchanturia et al., 2004), to personality traits (e.g., Moutafi et al., 2005; Reilly, 2012; Soubelet & Salthouse, 2011), or to levels of neuroticism (Jorm et al., 1993).

There are, of course, precautions that must be taken, as with any device and experimental method.

First, given the general visual size underestimation of the spheres we employed, it is necessary to have participants make visual matches with all spheres out of sight before the adaptation phase. This will allow to correctly calculate the size of the effects after adaption (Δ*T*).

Second, given the similarity between the size of the illusion reported by Kappers and Bergmann Tiest (2013) with their bisection method and the size, we reported for the nearest visual scale condition, and given also the results from a new experiment we conducted to measure the effect of the distance of a visual scale on crossmodal haptic size estimation (Daneyko, Maravita, & Zavagno, 2019), we suggest that the visual scale should be placed within hand reach from the observer (30–50 cm), a range which also minimizes the differences between the spheres actual sizes and their visual size evaluations. And of course, such distance should be kept constant for all participants.

Finally, it would be interesting to verify whether the *see what you feel method* could be adjusted to study other haptic aftereffects, for example, kinesthetic figural aftereffects (Köhler & Dinnerstein, 1947) and curvature aftereffects (Gibson, 1933; Vogels, Kappers, & Koenderink, 1996, 2001).

## Supplemental Material

sj-pdf-1-ipe-10.1177_2041669520944425 - Supplemental material for See What You Feel: A Crossmodal Tool for Measuring Haptic Size Illusions Click here for additional data file. Supplemental material, sj-pdf-1-ipe-10.1177_2041669520944425 for See What You Feel: A Crossmodal Tool for Measuring Haptic Size Illusions by Olga Daneyko, Angelo Maravita and Daniele Zavagno in i-Perception
