# Supplementary material for: See What You Feel: A Crossmodal Tool for Measuring Haptic Size Illusions
Source: Iperception. 2020 Aug 10;11(4):2041669520944425. doi: 10.1177/2041669520944425 (PMC7425280; doi:10.1177/2041669520944425)
Supplement: sj-pdf-1-ipe-10.1177_2041669520944425 - Supplemental material for See What You Feel: A Crossmodal Tool for Measuring Haptic Size Illusions [file sj-pdf-1-ipe-10.1177_2041669520944425.pdf]

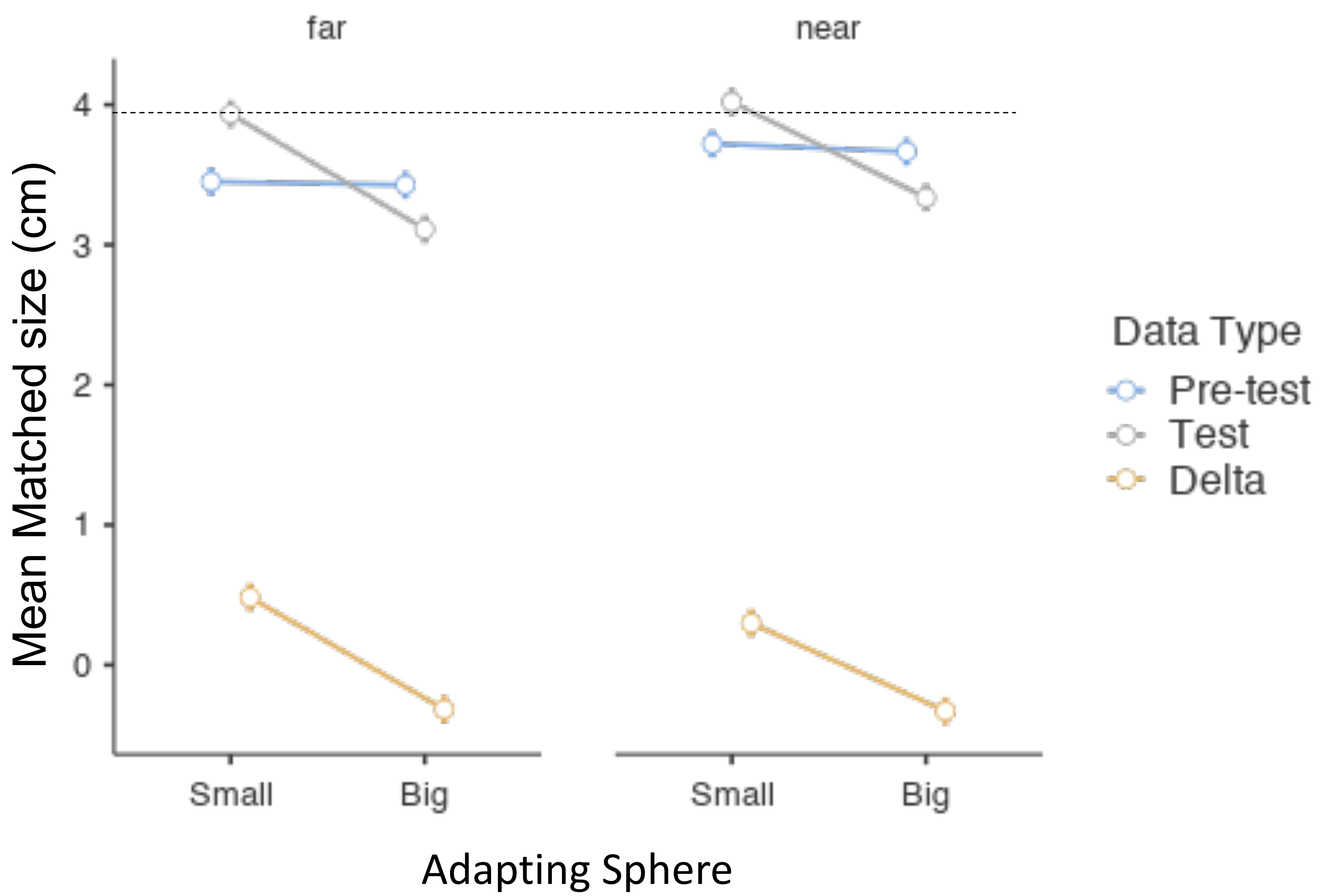

Supplemental Fig. 1: Mean matched size before adaptation (Pre-test) and after adaptation (Test). The graph shows also ΔT (Delta; ΔT=T-Pre-test). While looking simply at Test data one gets the impression that the illusion works only for a tests sphere in the hand adapted to a small sphere, ΔT shows the real dimension of the illusion, as the underestimations of the Test spheres prior to adaptation are taken into consideration. The dashed line shows the physical dimension of the Test spheres. Error bars are confidential intervals.
